# Feasibility of a Gelatin Temperature Sensor Based on Electrical Capacitance

**DOI:** 10.3390/s16122197

**Published:** 2016-12-20

**Authors:** Fernando Teixeira Silva, Brice Sorli, Veronica Calado, Carole Guillaume, Nathalie Gontard

**Affiliations:** 1 Joint Research Unit Agropolymers Engineering and Emerging Technologies, UMR 1208 INRA/SupAgroM/UMII/CIRAD, 2 Place Pierre Viala, 34060 Montpellier, France; c-guillaume@univ-montp2.fr (C.G.); gontard@univ-montp2.fr (N.G.); 2 Escola de Química, Universidade Federal of Rio de Janeiro, 21941-909 Rio de Janeiro, Brazil; calado@eq.ufrj.br; 3 Institut d’Electronique et des Systèmes, UMR CNRS 5214, Université de Montpellier, 34090 Montpellier, France; brice.sorli@ies.univ-montp2.fr

**Keywords:** sensor, gelatin, temperature control, electrical capacitance, meat cooking

## Abstract

The innovative use of gelatin as a temperature sensor based on capacitance was studied at a temperature range normally used for meat cooking (20–80 °C). Interdigital electrodes coated by gelatin solution and two sensors of different thicknesses (38 and 125 µm) were studied between 300 MHz and 900 MHz. At 38 µm, the capacitance was adequately measured, but for 125 µm the slope capacitance versus temperature curve decreased before 900 MHz due to the electrothermal breakdown between 60 °C and 80 °C. Thus, for 125 µm, the capacitance was studied applying 600 MHz. Sensitivity at 38 µm at 868 MHz (0.045 pF/°C) was lower than 125 µm at 600 MHz (0.14 pF/°C), influencing the results in the simulation (temperature range versus time) of meat cooking; at 125 µm, the sensitivity was greater, mainly during chilling steps. The potential of gelatin as a temperature sensor was demonstrated, and a balance between thickness and frequency should be considered to increase the sensitivity.

## 1. Introduction

Temperature measurements are very important for several types of industries. In the food industry, its monitoring is essential to guarantee the food’s safety; thus, it is a critical control point (CCP). The principal temperature sensors used are thermal resistor, thermal diode, and thermocouple [1], which are the most used because of their reliability and low cost [2]. Furthermore, the associated limitations related to meat cooking are: (1) the monitoring of only a few of the products in the oven and (2) the impossibility of monitoring the same product from heating to cooling steps (if they are performed separately) and during storage. These features open a window for tools that are able to control both production and distribution, possibilities that can be reached with wireless systems [3].

Combining a temperature sensor or indicator with a radio-frequency identification (RFID) tag can be the best choice for products in the chilling chain [4]. This application was reported by literature [3,5], but its use in other unit operations is scarce. However, for all of them, it is imperative for an adequate operation of temperature-sensitive processes.

Our research group has been studying biomaterials as environmental sensors, focusing on coupling them with RFID tags [6]. In our previous tests, gelatin was suitable as a sensor of temperature at high humidity (90% RH), the same variables normally used during heat treatment by meat industries. These projects follow the concept of the combination of biology and electronics required in modern biosensors, focusing on on-line measurements of important process parameters and microbial detection [7].

The temperature indicators normally are based on physical sensors; the use of biomaterial is an innovative proposal, based on the simplicity, low cost, and availability of renewable sources. Coupling it with a capacitive technique, which is also low cost and robust [8], may permit a cheap and efficient temperature sensor. The effectiveness of this technique is reported in several applications besides temperature [9]: volumetric concentration [8], moisture [10], DNA detection [11], and microbiological growth [12].

Gelatin has potential as a sensor because of its biocompatibility, biodegradability, low cost, and easy manipulation [13,14]. Besides, it is a material that is generally recognized as safe (GRAS), a necessary feature for food industry, and has high mechanical strength and characteristic of a stabilizing agent [15,16]. The chemical composition has a large number of polar functional groups that are beneficial to the polarization under an electric field [17]. As a hydrogel, it is able to absorb large amounts of water without not dissolving because of chemical or physical crosslinks and/or chain entanglements. Hydrogel responds to environmental changes, such as pH, temperature, and ionic strength [18].

There have been several reports combining gelatin and electrical properties [17,19,20,21,22,23] and its use as a biosensor, such as in biomedical applications and in the denaturation process [24,25,26,27,28,29]. However, the literature reporting gelatin as a temperature sensor is scarce. It was used as a protective and reducing agent for a visual physiological temperature sensor at room temperature [30].

The other advantage of gelatin is its potential as a biosensor because of different interactions (H-bonding, hydrophobic interactions, covalent bonding, etc.), leading to a biocompatibility with several quality markers (NH_2_, COOH, CONH_2_, OH, and SH) [31,32,33]; these properties demonstrate the great potential to control temperature and food spoilage after production and once in the market.

Engineering the bioelectrochemical sensing interface is crucial for improving its sensitivity [34]. In the literature, several methods have been used for this purpose, such as concentration of solution [35], polarization [36], addition of polyvinyl alcohol (PVA) [37], and addition of nanomaterials [34]. Thickness also influences the sensitivity [38]; indeed, this is the simplest variable to manipulate the sensitivity instead of adding components, such as before mentioned, which increases the complexity in preparing the sample, and homogeneity has also shown to influence the electrical properties [39]. Furthermore, working with thickness keeps the simplicity and low cost, which are qualities desirable for sensors.

The use of biomaterials to monitor temperature during processing in food industries, such as meat cooking, is a new concept, whose potential was already shown in our previous research (paper to be published soon). In this work, the use of gelatin was investigated in order to determine its feasibility as a temperature sensor. Our objectives were: (1) to study the influence of the layer thickness on the electrical capacitance sensitivity; (2) to evaluate its application under a meat-cooking protocol; and (3) to evaluate its stability for continuous use of the same sensor.

## 2. Material and Methods

The electrical properties of gelatin were explored, considering a temperature range of 20–80 °C and humidity of 90% RH—conditions normally used in meat cooking processing—as it allows achievement of water activity values close to those of the meat products (0.93–0.97). The frequency band studied was 300–900 MHz, focusing analysis at 868 MHz, which is the frequency used for the European ultrahigh frequency (UHF) RFID [6].

### 2.1. Differential Scanning Calorimetry (DSC)

The thermal analysis of the gelatin was carried out in a DSC from PerkinElmer (model Diamond) with an external refrigerating device (Intercooler II) and nitrogen as a purge gas system, with a flow rate of 20 mL·min^−1^. The temperature range was 25–170 °C, at a heating rate of 10 °C/min. The analyses were made in triplicate.

### 2.2. Scanning Electron Microscopy (SEM)

SEM analysis was carried out in an FEI Quanta 200 FEG. It is equipped with 50 mm^2^ X-Max Silicon Drift Detector, manufactured by Oxford Instruments. The sample was composed of a gelatin-coated interdigital capacitors (IDC) system on the SEM stubs.

### 2.3. Thickness

The average sample thickness was measured at the center and at four opposite positions by a handheld digital micrometer (0.001 mm). All samples were measured after coating the interdigital electrode. The experiments were made with 38 ± 1 µm (value close to the thickness of electrode—around 30 µm) and 125 ± 2 µm (reference value to the technical limit to cast the sample). Samples with thickness of 61 ± 1 µm and 84 ± 2 µm were used only for comparison.

### 2.4. Solution Preparation

Gelatin was used (Merck) with the following physicochemical composition: pH (3.8–7.6); SO_2_ (<0.005%); arsenic (<0.0001%); heavy metals (<0.001%); peroxide (<0.01%); phenolic preservatives (undetectable); sulphate dash (<20%); and grain size of 800 µm (99%). The concentration used was 10% w/v and the solution was prepared as shown by [40]. The bubbles dissolved in the solutions were removed by vacuum conditions.

### 2.5. Determination of Electrical Properties

Electrical properties were studied by a technique where sample is coated onto the IDC, with the response variable of electrical capacitance, determined according to Gevorgian model [41].

#### 2.5.1. Preparing Samples

The solution coated the surface of the interdigitated electrodes, with a circuit reference of 1 GHz (Cirly, France), by using the film applicator Coatmaster 510 (Erichsen, Germany), followed by a drying process at room temperature. A blank uncoated electrode was also used as a reference. IDC was used because of their versatile use in different environmental conditions and in a large range of frequencies [6].

#### 2.5.2. Determination of the Electrical Capacitance

**Figure 1:**
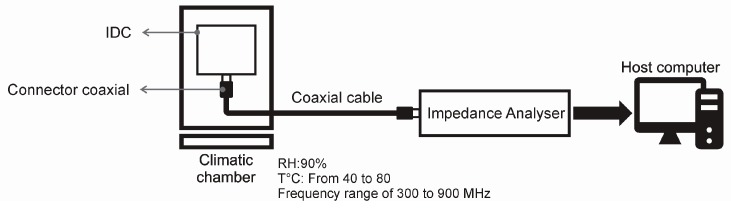
Experimental setup used for the electrical capacitance tests. IDC: interdigital capacitors.

The determination of the electrical capacitance was made in triplicate. The Impedance Analyzer HP 4191A RF was used at a frequency range of 300–900 MHz, and 500 mV for the oscillator voltage, which was linked to interdigital electrodes by a semirigid subminiature version A (SMA) coaxial cable (Amphenol Connex, Houten, The Netherlands) and the connector coaxial SMA 500 HM Solder SMA (Amphenol Connex) (Figure 1). The temperature and humidity were controlled by a climatic chamber (Espec, Osaka, Japan). The measurements were made at temperatures of 40–80 °C and at 90% RH, after stabilization of electrical capacitance. The time was calculated between the moment before changing the temperature and the moment just after starting the next stabilization of capacitance. An application test was made according to a protocol of meat cooking. The software used to record the results was LabVIEW (National Instruments, Redmond, WA, USA).

## 3. Results and Discussion

### 3.1. Effect of Temperature and Thickness on the Electrical Capacitance

**Figure 2:**
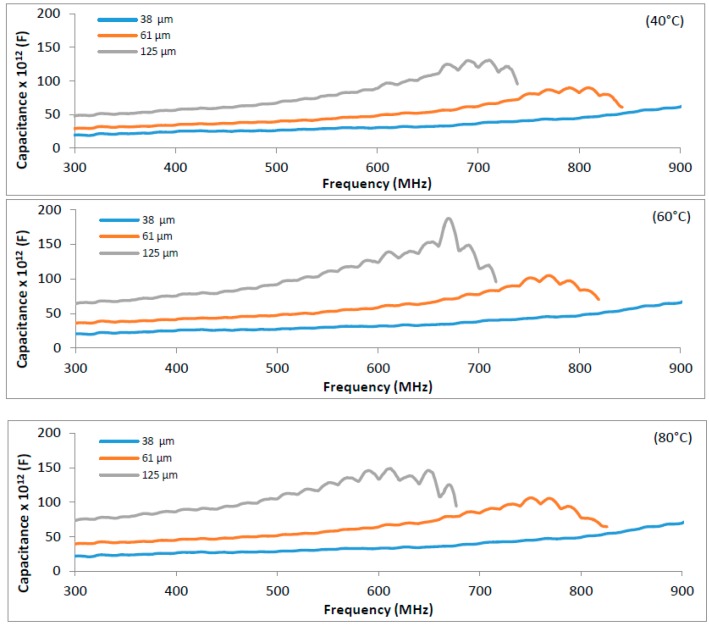
Influence of frequency (300–900 MHz) on the electrical capacitance of gelatin, with thickness of 38, 61, and 125 µm, for temperatures equal to 40 °C, 60 °C, and 80 °C. Experiments made in triplicate with coefficient of variation below 10%.

In our previous researches (paper to be published soon), using samples with thickness up to ~50 µm, the electrical capacitance was adequately measured at experimental conditions: 90% RH, 20–80 °C, and 300–900 MHz. This stability was also observed for a sample with a thickness of 38 µm and up to 900 MHz, but for the samples with greater thickness (61 µm and 125 µm), the curve of capacitance dropped before 900 MHz, to a value that was lower for higher temperature (Figure 2).

Each dielectric shows a characteristic behavior as a function of frequency and temperature [42]. The material can be ionized and become a conductor, as no dielectric material is a perfect insulator. A trace amount of electrical conduction is always present, especially at high electric field and/or elevated temperature. This tendency can be related to the dielectric strength according to the theories of Artbauer and electrothermal breakdown [43].

**Figure 3:**
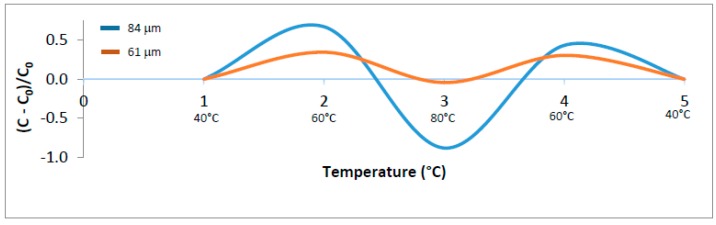
Effect of temperature on the capacitance of gelatin (expressed by (*C* − *C*_0_)/*C*_0_): thickness of 84 µm and 61 µm at 868 MHz and humidity of 90% RH: *C* (capacitance at 40 °C, 60 °C, 80 °C); *C*_0_ (capacitance at 40 °C).

Both theories are based on the influence of temperature, a variable that was assumed to explain the final stage of a breakdown process [44]. It can be seen that with a constant frequency (868 MHz), there was a conductor effect between 60 °C and 80 °C for the samples at 61 µm and 84 µm, which have finished after returning to 60 °C (Figure 3).

In the electrothermal breakdown, above a certain voltage, heat cannot be removed from the dielectric as rapidly as it is generated, which results in thermal breakdown [45]. Thus, the critical conductivity will be attained under a lower electric field when the temperature is higher. Consequently, the breakdown field decreases with the increase of temperature.

In Artbauer’s theory, the temperature dependence on the dielectric strength is understood in terms of the effect of temperature on the free volume and molecular relaxation process. When above the glass transition temperature, the molecules will be rearranged and there will be some free volume among them. The breakdown is influenced by the motion of charge carriers through voids in the polymer, arising from its free volume. The temperature increase leads to an increase of the available free volume and to larger void dimensions. Thus, the breakdown is happens more easily when temperature rises [42].

**Figure 4:**
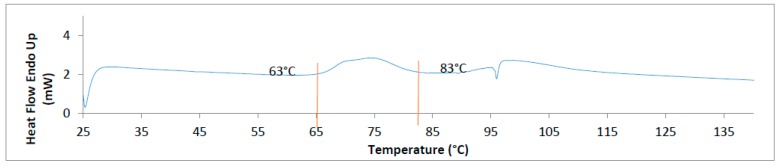
Result of differential scanning calorimetry (DSC) analyses of gelatin.

This theory was confirmed in DSC measurements of polypropylene foils that have revealed strong correlation between structural phase transitions at the same temperature regions, as it shows discontinuities in the breakdown strength [42]. The same was observed with the electrical properties of gelatin; the curve of capacitance dropped once between 60 °C and 80 °C (Figure 3) and it is quite the same band of temperature where the glass transition temperature (*T*_g_) started and finished, whose extrapolated value is 77.84 ± 0.13 °C (Figure 4).

Both theories mention and explain the breakdown temperature dependence that appeared only for thicker samples (61 µm and 125 µm) showing that thickness is also a variable that influences the electrothermal breakdown [46]. To work with thicker samples, the given frequency value obtained just before the capacitance starts decreasing may be used; in our case, this value was lower than 868 MHz, which it is the frequency used by the European System UHF RFID [6] (Figure 2).

The thickness affects the sensitivity, but there is a limit, considering loss of linearity at higher values [38]. This is supported by studies with IDC and polyimide as a sensor [47,48]. Thus, these results point out the necessity of having a good balance between thickness and frequency for the adequate use of a gelatin sensor. Based on our early studies with real permittivity of gelatin, 600 MHz was the highest frequency for better readability before reaching the resonant frequency value, and it was chosen to perform the following studies with sample with 125 µm.

A utilization of the sensor with a thick layer at 868 MHz may be considered in applications whose maximum temperature is lower than 60 °C, as mentioned earlier. In addition, although humidity was not a variable studied herein, in an assay at 45% RH and 868 MHz, the slope of the capacitance versus temperature curve did not decrease. This may permit the use of a thicker gelatin sensor in environments with reduced temperature ranges or with low humidity.

### 3.2. Hysteresis and Sensitivity

**Figure 5:**
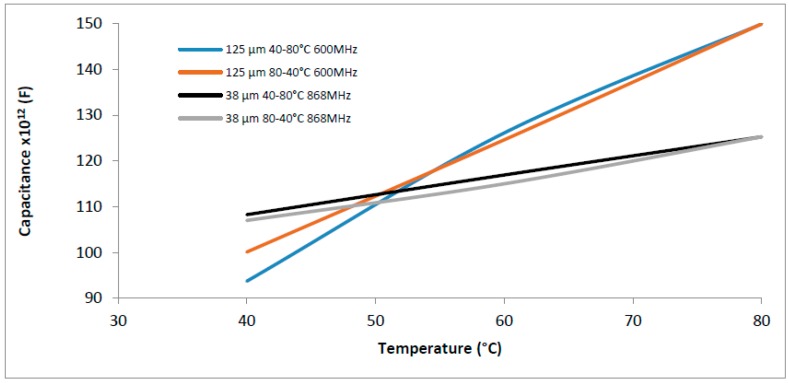
Hysteresis of gelatin from 40 °C to 80 °C and 90% RH for two thicknesses: 125 µm (600 MHz) and 38 µm (868 MHz). Experiments made in triplicate with coefficient of variation below 10%.

The hysteresis of gelatin samples with thicknesses of 38 µm and 125 µm are shown in Figure 5. The temperature range of 40–80 °C was studied, and once in this interval the instability of the capacitance measurements were normally observed.

Both curves (40–80 °C and 80–40 °C) for 38 µm were quite linear and, for 125 µm, the linearity of the curve was adequate for the rising temperature, but it changed for the descending one. The maximum hysteresis corresponds to 6% of capacitance at 40 °C (125 µm), but for all other points, it was below 2%, exhibiting a narrow hysteresis loop; this result is supported by literature [38]. In our previous tests, we observed the tendency of gelatin to stabilize at different levels for the same temperature (rising and descending), mainly for samples thicker than 50 µm.

The influence of thickness was also observed with regard to the time necessary to achieve stabilization of capacitance measurements under different temperatures. In general, the time for descending temperature rises, but for the 38 µm sample there was not a great variation of time, which was opposite for the 125 µm—once, the time quite doubled (Table 1). Further studies must be carried out in order to understand if the behavior observed in the descending temperature comes from the gelatin after *T*_g_ or from the climate chamber used in the tests.

The electrical capacitance depends on the thickness [12], which can be seen in Figure 5. The sample with thickness of 125 µm presented a curve with a higher slope, indicating a higher sensitivity. This relationship was calculated between 40 °C and 80 °C according to:

(1) S(m)=ΔC∕ΔT

where *S*—Sensitivity; ∆*C*—Quotient of the capacitance variation; ∆*T*—Quotient of the temperature variation.

The sensitivity for the sample with thickness of 125 µm was 0.14 ± 0.010 pF/°C (sample size = 3) and for that with thickness of 38 µm was 0.045 ± 0.009 pF/°C (sample size = 3), more than 3 times lower, showing that greater thickness leads to higher effectiveness of distinguishing the variation of temperature.

### 3.3. Meat Cooking Application

**Figure 6:**
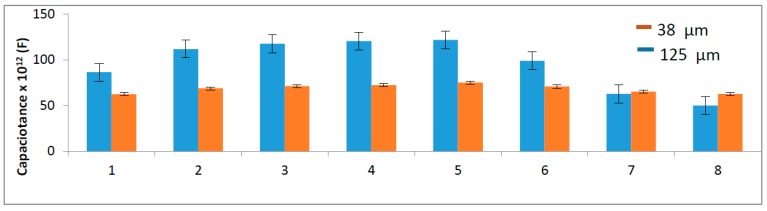
Use of a gelatin sensor for monitoring the heating processing in meat cooking: 90% RH, 125 µm (600 MHz) and 38 µm (868 MHz). Bars are: **1:** 40 °C for 30 min; **2:** 65 °C for 90 min; **3:** 70 °C for 60 min; **4:** 75 °C for 60 min; **5:** 80 °C for 60 min; **6:** 80–55 °C for 90 min; **7:** 55–27 °C for 120 min; **8:** 27–3 °C for 120 min. Error bar: standard deviation; sample size = 3.

The gelatin sensors (38 µm at 868 MHz and 125 µm at 600 MHz) were tested following the steps of meat cooking (Figure 6). It is clearly seen that the greater thickness (125 µm) led to a more distinguishable result, mainly in the cooling steps, the zone that permits effective food safety. Considering ready-to-eat products, such as ham and sausages, it is postulated that a cooling step, from 54.4 to 26.7 °C, no longer than 1.5 h, and from 26.7 to 4.4 °C, no longer than 5 h [49], is essential to reduce the activity of pathogenic microorganisms [9]. Both samples were able to show different electrical capacitances; however, with 125 µm, the system is more robust.

In the heating steps (2–5), the small difference of 5 °C has also resulted in a lower difference in the capacitance, which may limit the use of the gelatin sensor. Gelatin molecules have good polarization behavior because of a large number of polar functional groups. However, the presence of hydrogen bonds limits the mobility of these groups. To disrupt these bonds, it is necessary to improve the sensitivity, indicating possible blend with other molecules [17].

Applying thicker samples may be the opposite of the tendency to decrease the feature size of IDC design, by employing thinner dielectric [50]. However, for the use of gelatin as a sensor, a greater thickness was essential to increase the sensitivity, but it was not possible to use at 868 MHz because of the electrothermal breakdown.

### 3.4. Repeatability

**Figure 7:**
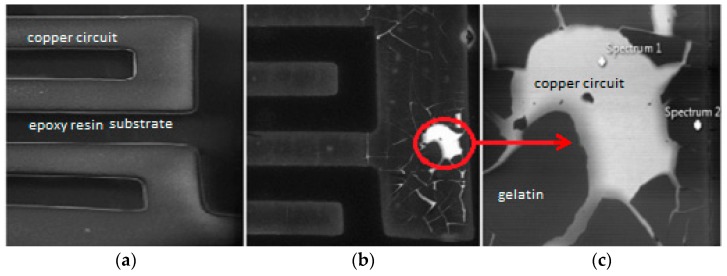
Images by SEM (scanning electronic microscopy) of the gelatin layer (38 µm) on the electrode: (**a**) electrode before use, 50×; (**b**) electrode after use, 50×; and (**c**) detail (400×) of the image from condition (**b**).

The repeatability of capacitance reading of the same gelatin sensor was investigated by using it thrice at three different temperatures (40 °C, 60 °C, and 80 °C), after storage at room temperature (around 25 °C) and humidity of 60%, approximately. The capacitance value obtained at the first measurement was considered as the reference. In general, the capacitance reduction was around 30% and 50% for the second and third times, respectively, as shown in Table 2. The capacitance obtained at each time and temperature was the result of the average of three measurements (repetitions) whose coefficient of variation was lower than 3%, showing data robustness. The explanations for the reduction can be shown in Figure 7, which shows the sensor before and after use. It can be clearly seen there is loss of material (Figure 7a,b). In the sensing region of the electrode (copper circuit), there is a loss of gelatin (spectrum 1) compared to spectrum 2, where there is also this material (Figure 7c). As the humidity used was high (90% RH), the gelatin was always wet, which facilitates adhesion to the electrode. However, with storage in low humidity (around 60%) and ambient temperature (25 °C), the film cracks, facilitating losses.

The capacitance readings were stable at high humidity, a situation that is reported as necessary to avoid loss of weight and consequential changes in electrical properties [26]. Indeed, the most important indicator that inhibits the continuous use of the sensor is not related to electrical measurement, but to the reduction of sensitivity. After storage at low humidity, for the third time, it was 0.019 ± 0.0001 pF/°C, more than two times lower than the first time (0.045 ± 0.009 pF/°C), as shown in Table 2.

We may conclude that it is possible to use the same biosensor in several heat treatments, as long as the humidity is kept high (above 90%). A wet storage condition could be considered [51] or the use of a wetting additive, but these conditions can facilitate food spoilage. Thus, refrigeration should be applied. However, these are possibilities that can increase the costs and reduce the simplicity of preparing and using gelatin. Then, the best option is to use a new sensor, after finishing an assay, as it is cheap.

## 4. Conclusions

The use of a gelatin sensor to provide accurate temperature measurements was ascertained for different thicknesses (38 µm and 125 µm) of films coated on interdigitated electrodes. For the sample with a thickness of 38 µm, the system was stable for all temperatures up to 80 °C and frequency range of 300–900 MHz. However, for the sample with greater thickness (125 µm), the temperature induced the electrothermal breakdown, limiting its use to 868 MHz. This phenomenon appeared around the temperature range of 60–80 °C, which coincides with the *T*_g_ zone of gelatin. In order to overcome the electrothermal breakdown, the experiments with the sample of 125 µm thickness were carried out at 600 MHz. The combination of greater thickness (125 µm) at 600 MHz in comparison with the sample of reduced thickness (38 µm) at 868 MHz resulted in a higher sensitivity and in a condition to better distinguish the different temperatures normally used in meat cooking, mainly in the cooling steps. It points to a good tradeoff between thickness and frequency, focusing to improve the electrical results. The gelatin sensor may be used several times under the same and continuous experimental conditions (90% RH and up to 80 °C) without variation in the capacitance. The reuse of the same gelatin sensor several times is not recommendable because it reduces the sensitivity as a result of mass loss after each use, when stored at low humidity. Gelatin sensors are feasible with tests under experimental conditions simulating parameters used in meat cooking. For a real production and application of the sensor, an interaction between gelatin and compounds of the food matrix must be considered.

## Figures and Tables

**Table 1:** Stabilization time (in minutes) of electrical capacitance of gelatin sensor with 38 µm (868 MHz) and 125 µm (600 MHz). All data are presented as average values ± standard deviations (sample size = 3).

Temperature (°C)	Time (min)	
	38 µm	125 µm
40–60	1.8 ± 0.1	15.1 ± 0.5
60–80	2.0 ± 0.1	14.3 ± 1.6
80–60	2.8 ± 0.2	34.5 ± 2.7
60–40	2.8 ± 0.1	42.8 ± 4.2

**Table 2:** Percentage of electrical capacitance reduction of the same gelatin sensor with 38 µm, at temperature range 40, 60, and 80 °C, 90% RH and 868 MHz.

Temperature °C	Reduction (%)		
	First Time	Second Time	Third Time
40	0	27 ± 1.2	46 ± 1.2
60	0	32 ± 1.5	47 ± 0.9
80	0	36 ± 0.5	48 ± 0.6
